# Familial Non-Medullary Thyroid Cancer Represents an Independent Risk Factor for Increased Cancer Aggressiveness: A Retrospective Analysis of 74 Families

**DOI:** 10.3389/fendo.2015.00117

**Published:** 2015-08-03

**Authors:** Martina Tavarelli, Marco Russo, Rosy Terranova, Claudia Scollo, Angela Spadaro, Giulia Sapuppo, Pasqualino Malandrino, Romilda Masucci, Sebastiano Squatrito, Gabriella Pellegriti

**Affiliations:** ^1^Endocrinology, Department of Clinical and Experimental Medicine, Garibaldi Nesima Medical Center, University of Catania, Catania, Italy; ^2^Endocrinology, Garibaldi Nesima Hospital, Catania, Italy; ^3^Surgical Oncology, Garibaldi Nesima Hospital, Catania, Italy

**Keywords:** thyroid, thyroid cancer, familial thyroid cancer, thyroid cancer prognosis, familial non-medullary thyroid cancer

## Abstract

**Objectives:**

To assess whether familial non-medullary thyroid cancer (FNMTC) represents an independent risk factor for increased aggressiveness of the tumor, as concern as the clinical presentation and the long-term follow-up in respect of sporadic differentiated thyroid cancer (SDTC).

**Design:**

Retrospective study; 1976–2014.

**Patients and Methods:**

Seventy-four FNMTC families (151 affected individuals): family relationship and number of affected family members were evaluated. Clinical and histopathological features and outcome were compared to that of 643 SDTC patients followed in the same period according to the same institutional protocols. Median follow-up was 57.7 months (range 12–136) in FNMTC and 59.7 (range 15–94.6) in SDTC patients.

**Results:**

Three cases occurred in 3 families and 2 cases in the other 71. F:M was 3.7:1 in FNMTC and 4.3:1 in SDTC (NS). The family relationship was siblings in 62.2%. Mean age at diagnosis was lower in FNMTC than in SDTC (*p* < 0.005). Papillary/follicular histotype distribution was similar (86%). Papillary tumors were more frequently multifocal in FNMTC (*p* = 0.004) and with lymph-node metastases (*p* = 0.016). Disease-free survival (DFS) was shorter in FNMTC vs. SDTC (*p* < 0.0001) with 74.8 vs. 90.8% patients free of disease at the last control *(p* < 0.005). Three patients died in FNMTC group vs. 1 in SDTC (*p* = 0.02).

**Conclusion:**

Familial non-medullary thyroid cancer displays distinct characteristics as earlier age of onset and increased aggressiveness at diagnosis and a higher rate of persistent/recurrent disease and mortality with a shorter DFS in respect with SDTC. FNMTC patients, therefore, should be followed accurately. As the specific gene (or genes) responsible for susceptibility for FNMTC has not yet been identified, a low frequency periodic screening of relatives DTC patients may be useful to identify FNMTC patients at early stage of disease.

## Introduction

Familial non-medullary thyroid cancer (FNMTC) represents approximately 3–7% of all thyroid cancers that originate from thyroid follicular epithelial cells (1).

Since the genetic alterations responsible for this disease are not known, FNMTC is defined as the condition in which two or more first-degree relatives are affected by thyroid cancer in the absence of a known familial syndrome.

However, due to the high prevalence of differentiated thyroid carcinoma (DTC), it is estimated that when two members of the same family are affected, there is a 53% probability that the disease has a familial origin but also a 47% probability that it is sporadic. When three or more family members are affected, the probability rises to 99.9% (2).

As already mentioned, to be defined familial, FNMTC should not represent the phenotypic expression of a genetic familial syndrome. There are, in fact, familial cancer syndromes regarding non-thyroid tumors where thyroid carcinoma may also be present. These include the Cowden syndrome (multiple hamartomas in the skin and mucosa and cancers in various organs and tissues, such as the breast, thyroid, and uterus), the familial adenomatous polyposis (FAP) characterized by multiple and diffuse adenomatous polyps in the colon, osteomas, epidermoid cysts, desmoid tumors, hamartomas of the upper digestive tract, congenital hypertrophy of retinal pigment epithelium, hepatoblastoma, and the Carney complex (pigmentation of the skin and mucous membranes, mucosal lesions, and tumors of the endocrine glands, such as pituitary and adrenal adenomas, Sertoli and Leydig cell tumors, and thyroid carcinoma), the Werner syndrome (characterized by premature aging, bilateral cataracts, gray hair, skin atrophy, and cancer in different organs including the thyroid), and finally syndromes in which thyroid cancer is associated with clear cell renal carcinoma (3, 4).

Many studies suggest that FNMTC is a defined entity with early cancer onset and aggressive features relative to sporadic thyroid cancer but the topic remains controversial (5). The present study aims to assess whether FNMTC represents a homogeneous biological entity that is significantly different from sporadic differentiated thyroid cancer (SDTC). In a continuous series of FNMTC patients followed at our Thyroid Clinic we evaluated, the number of affected family members, the modality of the cancer transmission, the onset characteristics (incidence for gender and age and clinical presentation), histopathological features, and long-term outcome. Data from FNMTC patients were compared to a control group of SDTC patients.

## Patients and Methods

We retrospectively analyzed a consecutive series of 4120 thyroidectomized DTC patients in the 1976–2014 period, all followed at the Thyroid Clinic, Division of Endocrinology, Garibaldi Medical Center in Catania, Italy.

Among this series, we identified 264 (6.4%) patients with a positive family history that were classified FNMTC because at least one first-degree relative was affected by confirmed thyroidal cancer of follicular origin.

Only patients having all family members with FNMTC in continuous follow-up in our Center were included: 151 patients belonging to 74 families.

All patients underwent total thyroidectomy, with a central and/or lateral-cervical lymph-node dissection in 53 cases (35.1%).

Tumors were staged according to the VII TNM edition (6): T (the extent of the primary tumor) and N (regional lymph-node metastases) were assessed on the basis of pathological examination, and M (distant metastases) according to the first post-surgical 131I-whole-body scan (131I-WBS).

After surgery, radioiodine (1110–3700 MBq) was administered to 131/151 patients (86.7%) who met one or more of the subsequent criteria: tumor size >1.0 cm, tumor extension to soft tissues adjacent to the thyroid gland, multifocal tumor, and/or nodal metastases. Follow-up procedures included neck ultrasound and serum thyroglobulin (Tg) after either L-T4 withdrawal or recombinant human TSH stimulation.

Persistent/recurrent disease was defined by one or more of the following criteria: (1) serum Tg, either under suppressive L-T4 therapy or after TSH stimulation, at detectable levels, as defined by the assay sensitivity limits at the time of measurement; (2) evidence of metastatic lymph nodes identified at neck ultrasound and confirmed by FNAB with Tg measurement in the aspirate washout; (3) positive 131I-WBS. Computed tomography, magnetic resonance imaging, bone scan, and positron emission tomography were performed when appropriate in patients with persistent/recurrent disease.

Data in FNMTC patients were compared to those obtained in a control group of 643 SDTC patients matched at 1:4 ratio for the long-term follow-up. In the control group, post-operative radioiodine treatment was administrated in 390 cases (60.6%).

Median follow-up was 57.7 months (range 12–136) in FNMTC and 59.7 (range 15–94.6) in SDTC patients.

### Ethical approval

All procedures involving human participants were in accordance with the ethical standards of institutional research committee and with the Helsinki declaration as revised in 2013. Informed consent of the present retrospective study was waived.

### Statistical analysis

Quantitative data were compared using the *t*-test, and qualitative data were compared by the chi-square analysis. The disease-free survival (DFS) curves were determined by Kaplan–Meier curves. *p* values <0.05 were considered statistically significant.

## Results

### Familial characteristics and the mode of transmission of tumors in patients with FNMTC

Seventy-one (95.9%) of the 74 families with FNMTC had two affected family members, and three families patients (4.1%) had three affected family members. The transmission of the disease was maternal in 40 cases from 20 families and paternal in 11 cases from 5 families. In 94 cases (47 families), the disease was present in brothers and sisters. In six cases (two families) in addition to the first-degree also a second-degree relative with FNMTC was identified (two sisters and their maternal aunt and brother, sister, and her daughter affected in the two families, respectively) (Table 1).

**Table 1:** **Family relationship of patients with FNMTC**.

	Families (74) *n*.%	Patients (151) *n*.%
Paternal	5 (6.8)	11 (7.3)
Maternal	20 (27.0)	40 (26.5)
Sibling (brother-sister)	47 (63.5)	94 (62.2)
Other	2 (2.7)	6 (4.0)

### Characteristics of patients with FNMCT and SDTC

Table 2 shows the characteristics of patients with FNMTC and SDTC.

**Table 2:** **Characteristics of FNMTC and SDTC patients**.

	FNMTC (151)	SDTC (643)
Gender (F/M) ratio	119/32	521/122
Age (years)	3.7/1	4.3/1
Mean ± SD	45.4 ± 13.5	48 ± 13.7*
range	17.1–81.5	16.8–82.6

*p < 0.005.

Of the 151 FNMTC patients studied, 119 (78.8%) were females (F) and 32 (21.2%) were males (M), with an F/M ratio of 3.7/1; in the control group (643 patients with SDTC), there were 521 F (81.0%) 122 M (19.0%), with an F/M ratio of 4.3/1 (*p* = 0.57).

The median age at diagnosis was 45.4 ± 13.5 years for FNMTC (range 17.1–81.5) and 48.0 ± 13.7 years (range 16.8–82.6) for SDTC (*p* < 0.005).

### TNM staging and histopathological features of thyroid carcinoma in FNMTC and SDTC

Table 3 shows the distribution of histological types in the two groups studied.

**Table 3:** **Histotypes of FNMTC and SDTC**.

Histotype	FNTMC (151) *n*.%	SDTC (643) *n*.%
Papillary classic and follicular variant	130 (86.1)	558 (86.8)
Diffuse sclerosing variant	4 (2.6)	15 (2.3)
Tall cell variant	3 (2.0)	29 (4.5)
Papillary cistyc variant	1 (0.7)	0 (0)
Follicular	10 (6.6)	23 (3.6)
Hurtle Cell	3 (2.0)	18 (2.8)

The ratio between the papillary or follicular histological type was in favor of the papillary and its variant in familial compared to sporadic thyroid carcinoma, which showed no statistically significant difference (11.6/1 vs. 14.7/1, respectively).

Table 4 shows the histopathologic features of tumors in both groups, including size, multifocality, bilaterality, and extrathyroidal extension.

**Table 4:** **Histopathologic features of FNMTC and SDTC**.

	FNMTC (151)	SDTC (643)
Tumor size (cm)	1.5 ± 1.2	1.5 ± 1.3
Mean ± SD range	0.1–7.0	0.4–6.0
Multifocality (%)	69 (45.7)	214 (33.2)*
Bilaterality (%)	48 (31.7)	158 (24.5)
Extrathyroidal extension (%)	33 (21.8)	126 (19.6)

*p = 0.004.

In patients with FNMTC, the medium tumor size was 1.5 ± 1.2 cm (range 0.1–7.0). In addition, the tumor was multifocal in 69 cases (45.7%), bilateral in 48 (31.7%), and with extrathyroidal extension in 33 cases (21.8%).

In the control group, tumor medium size was 1.5 ± 1.3 cm (range 0.4–6.0), multifocal in 214 cases (33.2%), bilateral in 158 (24.5%), with extrathyroidal extension in 126 (19.6%).

The percentage of multifocal tumors in patients with FNMTC compared to patients with SDTC was highly significant (*p* = 0.004).

No significant differences were found regarding bilateral involvement or extrathyroidal extension.

Table 5 shows the TNM staging of FNMTC and SDTC. Any significant differences among stages were observed in the two groups of patients (Table 6).

**Table 5:** **TNM (VII ed.) staging of FNMTC and SDTC**.

	FNMTC (151) *n*.%	SDTC (643) *n*.%
pT1	86 (57.0)	418 (65.0)
pT2	13 (8.6)	79 (12.3)
pT3	38 (25.2)	144 (22.4)
pT4	1 (0.6)	2 (0.3)
pTx	13 (8.6)	0 (0)
N1	40 (26.4)	113 (17.5)*
M1	4 (2.6)	8 (1.2)

*p = 0.016.

**Table 6:** **TNM staging (VII ed.) of FNMTC and SDTC**.

	FNMTC (151) *n*%	SDTC (643) *n*%
Stage I	111 (73.5)	486 (75.6)
Stage II	8 (5.3)	43 (6.7)
Stage III	22 (14.6)	94 (14.6)
Stage IV	7 (4.6)	20 (3.1)
Not evaluable	3 (2.0)	0 (0)

In FNMTC, we found a lower proportion of microcarcinomas than in sporadic thyroid cancer (60/151 vs. 316/643, respectively, *p* = 0.038). No significant difference was found among the various groups’ pT, while there was a more significant presence of lymph-node metastases in familial tumors (40/151 vs. 113/643, respectively, *p* = 0.016).

### Outcome and prognosis

We evaluated, in both groups of tumors, the DFS and the number of patients without persistent/recurrent disease at the last control visit.

**Figure 1:**
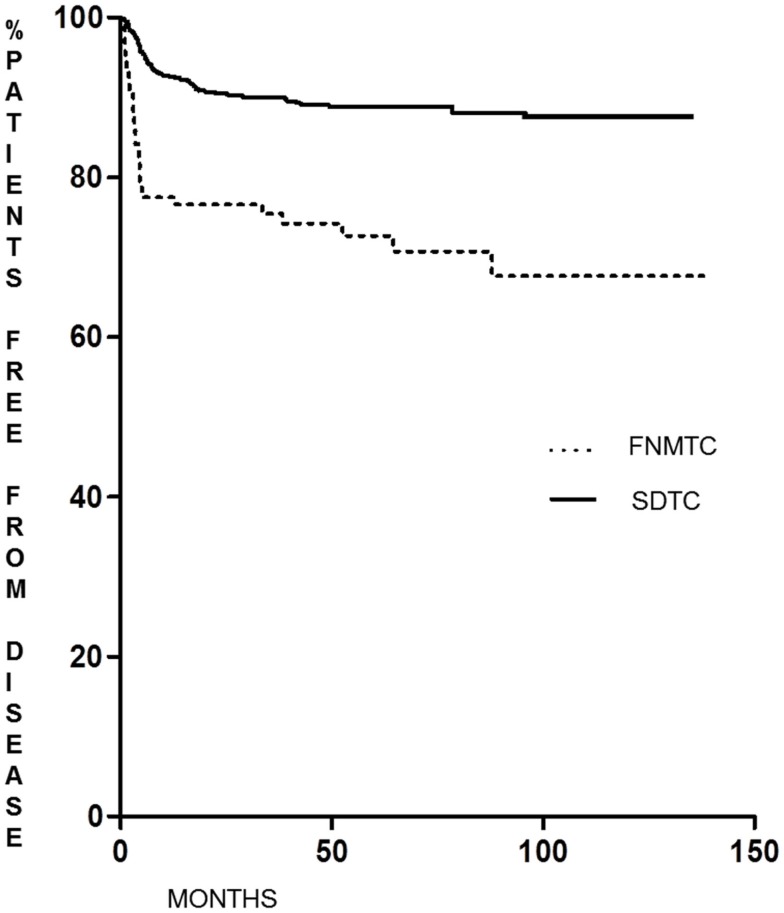
**Disease-free survival in FNMTC vs. SDTC patients**.

**Figure 2:**
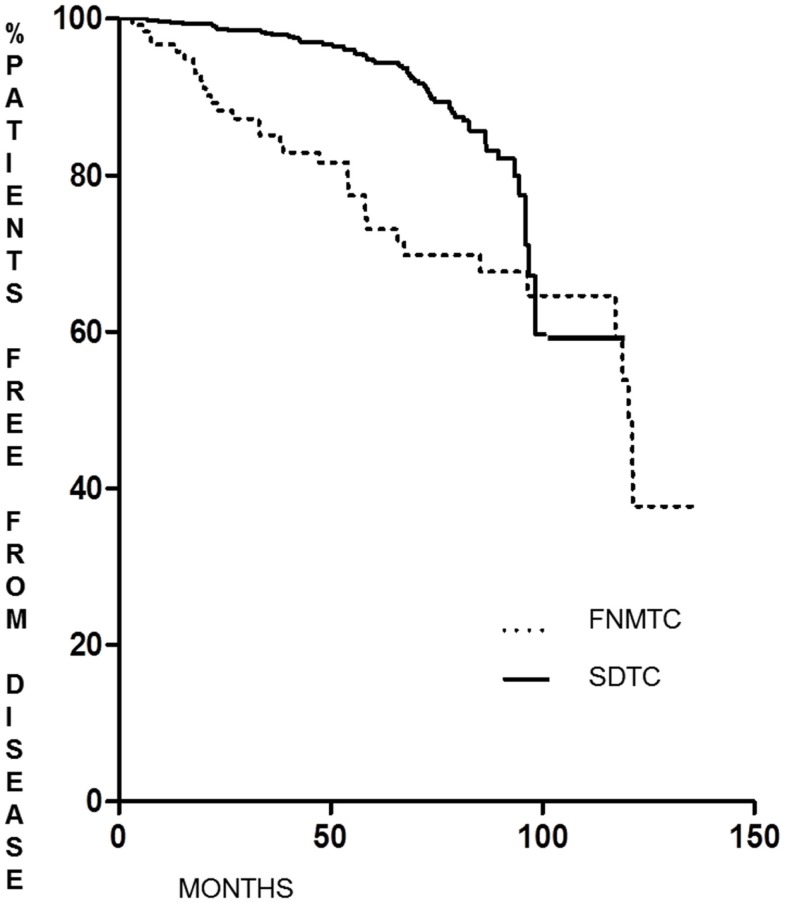
**Disease-free status at last control visit in FNMTC vs. SDTC patients**.

As shown in Figure 1, the DFS of patients with FNMTC [median 33.8 months (range 6–136)] was significantly shorter (*p* < 0.0001) than that observed in patients from the control group [median 55.9 months (range 6–95)]. In FNMTC patients, at the last control visit, we observed a lower percentage of cases free from disease (*p* < 0.0001) than we observed in the control group (Figure 2). In particular, 113/151 (74.8%) FNMTC patients were disease free and 38 (25.2%) showed persistent disease. Three patients died for the disease. Concerning SDTC patients, 584 (90.8%) were disease free and 59 (9.2%) had persistent disease at the last control visit; 1 patient died (Table 7).

**Table 7:** **Disease status al last control visit in FNMTC and SDTC patients**.

	FNMTC (151) *n*.%	SDTC (643) *n*.%
Disease free	113 (74.8)	584 (90.8)*
Persistent disease	38 (25.2)	59 (9.2)
Lymphnodes	8 (21.1)	10 (16.9)
Distant metastases	8 (21.1)	12 (20.3)
Local and distant metastases	4 (10.5)	0 (0)
Only detectable thyroglobulin	18 (47.3)	37 (62.8)

*p < 0.001.

Concerning familial microcarcinomas (60 patients), we did not find any significant differences with respect to the control group (316 patients), either regarding the DFS (6 recurrences in FNMTC vs. 14 in SDTC) or the percentage of disease-free patients at the last control visit (persistence of disease in 3 FNMTC patients vs. 13 in SDTC).

Regarding familial microcarcinomas, which according to the TNM system can be classified as T1a N0/NX M0 (29 cases), there was no significant difference for DFS (*p* = 0.07) or for the percentage of disease-free patients at the last control visit (*p* = 0.23) compared to patients with SDTC (189 cases).

**Figure 3:**
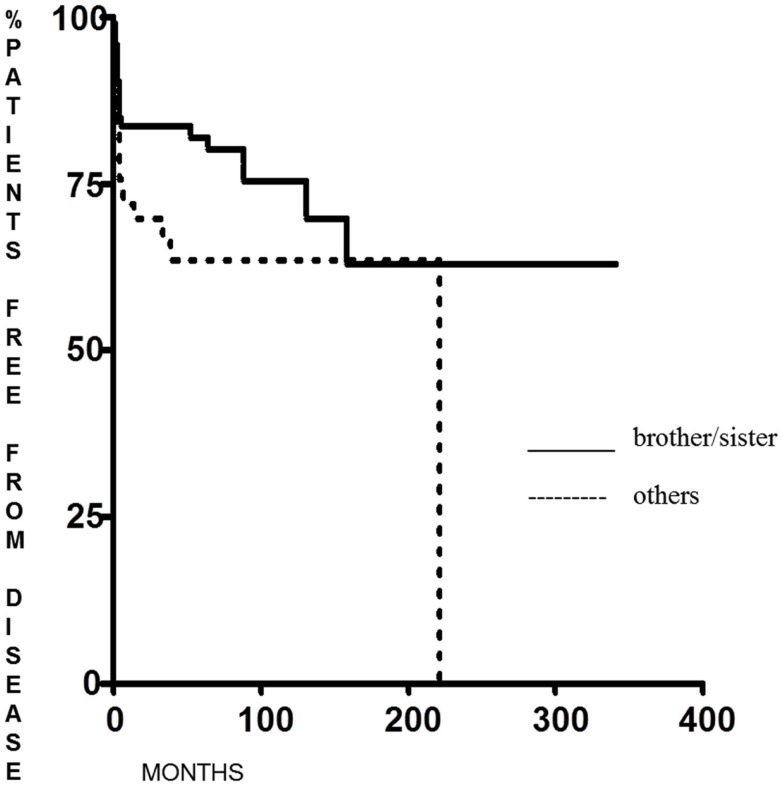
**Disease-free survival in FNMTC patients with brother/sister tumor transmission vs. patients with other relationship**.

**Figure 4:**
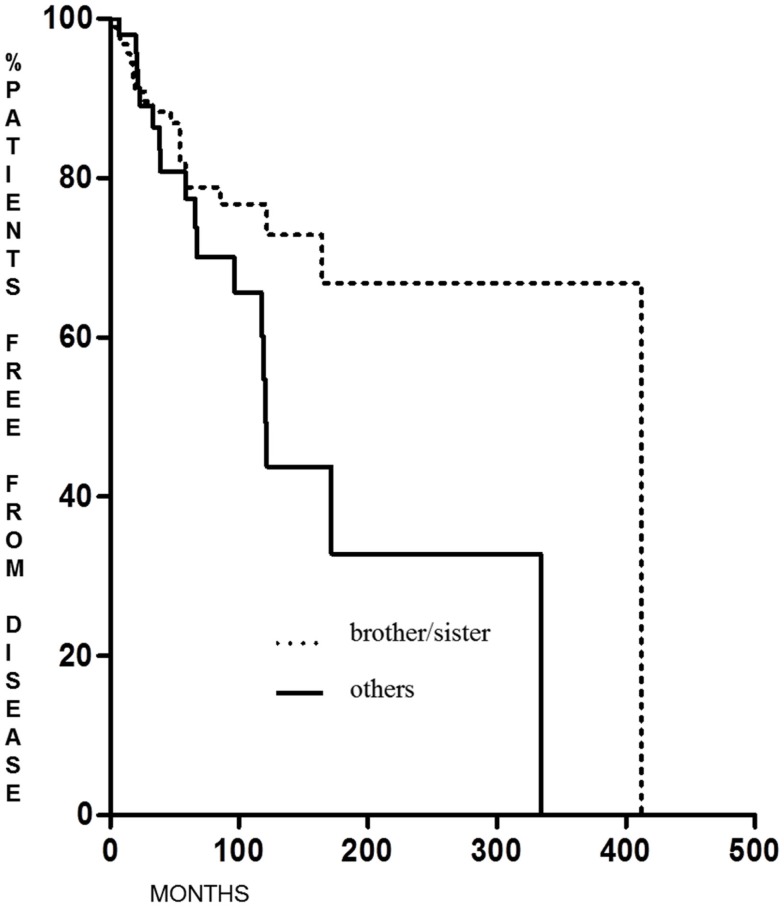
**Cured patients at last control visit in FNMTC with brother/sister tumor transmission vs. patients with other relationship**.

Familial non-medullary thyroid cancer with maternal transmission was not more aggressive than other FNMTCs (*p* = 0.23 for DFS and *p* = 0.28 for the percentage of patients free of disease at the end of follow-up); on the contrary, we found that DFS was significantly longer (*p* = 0.038) in patients in whom the family relationship was brother–sister than in other relationships [62.6 months (range 6–341 months) and 12 months (range 6–222 months), respectively] (Figure 3), with a greater percentage of patients free from disease at the last control visit (*p* < 0.005) (Figure 4).

## Discussion

Familial non-medullary thyroid cancer is a rare entity that accounts for approximately 3.3–6.9% of all thyroid cancer cases (7).

The clinical behavior of FNMTC is still controversial, including the rate of persistence/recurrence and patient survival compared to sporadic thyroid cancer. In our study, the most frequent mode of cancer transmission was between brothers and sisters (62.2%), followed by maternal transmission (26.5%) and paternal transmission (7.3%) and in two families (4%) in addition to the first-degree also a second-degree relative with FNMTC was identified. There are few data regarding the mode of transmission in the literature, with the exception of the study by Rumianteseva et al. in which, in a series of 48 patients with FNMTC belonging to 24 families, was found a greater frequency of maternal transmission (75.0%) (8).

In our study, FNMTC is diagnosed earlier than SDTC (45.4 ± 13.5 vs. 48.0 ± 13.7 years to diagnosis). The early onset of FNMTC may be due to an early diagnosis because of the familial nature of the disease or to the phenomenon of anticipation typical of familial cancers which, according Capezzone et al. (9, 10), derives from an alteration of the telomere–telomerase complex, which is characterized by short telomeres and increased amplification and expression of the gene coding for telomerase reverse transcriptase (hTERT) (11). This finding and the more aggressive presentation at diagnosis agree with Linch’s hypothesis that FNMTC tends to appear earlier in members of families where the transmission involves first-degree relatives. Other studies have shown similar findings: in the study by Loh (12), FNMTC was diagnosed 10 years earlier than sporadic thyroid carcinoma; similar results were recently reported by different authors (13–15). According to Mc Donald, age at presentation in FNMTC patients was not different when compared to the sporadic form (16).

From a histological viewpoint, FNMTC is indistinguishable from sporadic forms of well-DTC, and these data were confirmed in our study. Regarding the histopathological features of the tumor, we found a greater number of multifocal tumors in FNMTC compared to the control group. This feature, as reported in many studies, constitutes one of the peculiarities of this form of cancer. The largest study regarding the characteristics of patients with FNMTC is from Japan. Uchino et al. examined 6,458 patients and identified 258 cases of FNMTC, with a median age at diagnosis of 49 years, and although the tumor size at diagnosis was not different compared to sporadic cancers, multifocality was observed more frequently (42 vs. 30%) (17). According to Clark et al., FNMTC is multifocal in 93% and bilateral in 43% of cases while in sporadic thyroid cancer, multifocality is observed in 20–32% of cases and bilateral foci are detected in 19% (1).

Similar data were found by Alsanea (18) and Ito (19), who showed that FNMTC was multifocal in 46 and 38%, respectively. Other recent studies confirmed these findings (13, 14, 20, 21).

In our study, FNMTC more frequently exhibited nodal involvement at diagnosis compared to sporadic forms, although we did not observe a major incidence of extrathyroidal tumors.

This is in agreement with other data in the literature: in 1995, Grossman documented an incidence of lymph-node metastases at diagnosis in 57% of patients with FNMTC vs. 38% of patients with sporadic thyroid carcinoma (22). Similar data were reported in the study of Alsanea (18) and Mazeh (13), but these data were not confirmed by Ito (19), Uchino (17), and Moses (15).

Regarding clinical evolution and prognosis, we found a significant reduction in DFS and a worse prognosis in FNMTC than in SDTC. In 1995, Grossman documented a recurrence rate of 50% in patients with FNMTC vs. 15% in patients with SDTC (22). Alsanea confirms these data and reports a lower rate of survival in patients with FNMTC, with a recurrence of 44 vs. 17% in the control group (18). In Uchino et al. series, FNMTCs showed no significant increase of local invasion or lymph-node metastases at diagnosis compared to SDTC patients but had a significant recurrence rate (16.3 vs. 9.6% *p* = 0.0005) with a DFS significantly shorter (*p* = 0.004) (17).

Patients with differentiated thyroid microcarcinoma typically have an excellent prognosis. Lupoli et al., evaluating a series of tumors in patients with familial and sporadic microcarcinoma, showed that in familial forms, the tumor was multifocal in 71% of cases (vs. 19% in sporadic forms) and bilateral in 43% (vs. 19%), with high frequency of lymph-node metastases (57 vs. 28%) and vascular invasion (43 vs. 5%). The recurrence rate in these patients was significantly higher (43 vs. 5%), and one patient died of widespread metastasis (23). This study shows that the aggressive nature of FNMTC persists in very small tumors. Others studies do not confirm these findings (24, 25). According to the data emerging from our study, 60 FNMTC patients with microcarcinoma did not show a significant difference compared to 316 patients in the microcarcinoma group control, either regarding DFS or the percentage of patients free from disease at last control visit.

It is now widely recognized that familial cancers are more aggressive than sporadic ones, even if it is difficult to separate the two forms, especially when only two family members are affected; in fact, in this case, there is a 69% probability that the cancer is familial, but it might also be a sporadic thyroid cancer (26).

In families where three or more family members are affected and where genetic alteration is almost certain, the high aggressiveness of the tumor is clearly demonstrated by the prognosis and the final outcome, as showed in the study by Triponez et al. (2). He showed that survival is reduced when the diagnosis of FNMCT is made before the family pathology is recognized compared to patients who have a known family history of thyroid cancer.

This suggests that early diagnosis and treatment are very important to improve the quality of life and survival of patients with FNMTC. Therefore, pre-screening is recommended in patients who have family members with FNMTC (approximately when they are 10 years younger than the affected family members) (1).

A recent meta-analysis of Wang et al. reporting 12 studies with a total of 12,741 patients confirmed that FNMTC shows more aggressive biological behaviors with younger age at diagnosis, high risk of multifocal, bilateral, extrathyroidal tumor, and lymphnode metastases at diagnosis and has an increased rate of recurrence and decreased DFS in comparison to SDTC (27).

Our study also showed that the siblings of a family member with FNMTC have a better prognosis than off-spring of parents with FNMTC, and these data were similar to that reported by Park et al. (14).

Regarding the type of treatment, all patients with FNMTC should undergo total thyroidectomy to avoid, in genetically predisposed patients, the proliferation of residual tumor tissue. Due also to the high incidence of lymph-node metastasis to the central neck (level VI) at the time of diagnosis, central lymph-node dissection should be considered for these patients. Involvement of lateral neck cervical lymph nodes should be assessed pre-operatively.

Furthermore, because FNMTC has a high recurrence rate, which is an important cause of reduced patient survival, many experts recommend that all patients should undergo I131 radioactive iodine treatment and post-surgery L-T4 suppressive therapy.

At present, FNMTC follow-up guidelines are not available, but due to the points discussed above, surgical and post-operative treatment and follow-up should be planned because of the aggressiveness of these tumors. It will be useful to separate these patients, even if belonging to the same class of risk, according to the presence or absence of familial cases. Therefore, the majority of patients should undergo radioiodine ablation independently of tumor size to achieve complete ablation of residual thyroid tissue (1, 17).

The genetic basis of FMNTC remains unknown, but it is believed that the genetic alterations are transmitted as an autosomal dominant incomplete penetrance and variable expressivity.

Some of the chromosomal loci identified could play a role in the development of cancer, but none of them seem sensitive to propose a genetic screening test for high risk-families.

In conclusion, our study showed that:

1. Familial non-medullary thyroid cancer is found more frequently in siblings.
2. Familial non-medullary thyroid cancer is diagnosed at an earlier age than sporadic thyroid carcinoma; it is less frequently a microcarcinoma but more often shows multifocal foci and lymph-node metastases at diagnosis.
3. Familial non-medullary thyroid cancer has a worse prognosis than SDTC, with a disease-free time significantly lower than that observed in patients with sporadic thyroid cancer.
4. Siblings of individuals with FNMTC have a better prognosis than FNMTC with other relationships ones.

Further studies are still needed to define the best management for FNMTC. Because there is not a genetic test to identify at-risk patients, an early screening of patients who have family members with FNMTC is essential to identify the tumor at an early stage.

## Conflict of Interest Statement

The authors declare that the research was conducted in the absence of any commercial or financial relationships that could be construed as a potential conflict of interest.
